# One new species and one new record for the genus *Ninodes* Warren from China (Lepidoptera, Geometridae, Ennominae)

**DOI:** 10.3897/zookeys.679.12547

**Published:** 2017-06-08

**Authors:** Xinxin Li, Dayong Xue, Nan Jiang

**Affiliations:** 1 Key Laboratory of Zoological Systematics and Evolution, Institute of Zoology, Chinese Academy of Sciences, Beijing 100101, China; 2 University of the Chinese Academy of Sciences, Beijing 100049, China

**Keywords:** Cassymini, diagnosis, genitalia, morphology, *N. albarius*, *N. quadratus*, taxonomy

## Abstract

A new species of the genus *Ninodes* Warren, *N. quadratus* **sp. n.**, is described from China and compared with related species, based on numerous museum specimens. *N. albarius* Beljaev & Park, 1998, described from Korea, is newly recorded for China. Illustrations of external features and genitalia for each species of *Ninodes* are presented.

## Introduction

The genus *Ninodes* was described by Warren (1894) on the basis of *Ephyra splendens* Butler, 1878 from Japan, and is currently placed in the tribe Cassymini within the subfamily Ennominae. *Ninodes* Warren is a small genus with four recognized species, *N. splendens* (Butler, 1878), *N. albarius* Beljaev and Park, 1998, *N. flavimedia* Warren, 1907 and *N. watanabei* Inoue, 1976 (Beljaev and Park 1998; Scoble 1999). The species of *Ninodes* are mainly distributed in East Asia, but only *N. splendens* has been recorded from China (Prout 1915; Wehrli 1939; Heppner and Inoue 1992; Xue 1992, 2001).

The purpose of this paper is to describe one new species, *N. quadratus* sp. n., and to provide a differential diagnosis of the new species and its relatives, to report *N. albarius* as newly recorded for China, and to provide illustrations of external features and genitalia for all species of *Ninodes* to facilitate identification of the species belonging to this genus.

## Materials and methods

Specimens of *Ninodes* were mainly taken from collections of the Institute of Zoology, Chinese Academy of Sciences, Beijing, China (**IZCAS**), the Museum National d’Histoire Naturelle, Paris, France (**MNHN**), the Natural History Museum, London, UK (**NHM**), the Senckenberg Museum für Tierkunde, Dresden, Germany (**SMTD**) and the Zoologisches Forschungsmuseum Alexander Koenig, Bonn, Germany (**ZFMK**). A further institution cited as type depositary is the Laboratory of Insect Taxonomy, National Institute of Agricultural Science and Technology, Suwon, Korea (**NIAST**).

Terminology for wing venation follows the Comstock-Needham System (Comstock 1918), and that for the genitalia is based on Pierce (1914, reprint 1976), Klots (1970) and Nichols (1989). Photographs of the moths were taken with digital cameras. Composite images were generated using Auto-Montage software version 5.03.0061 (Synoptics Ltd). The illustrations were compiled using Adobe Photoshop software.

## Taxonomy

### Genus Ninodes

Taxon classificationAnimaliaLepidopteraGeometridae

Warren, 1894

Ninodes Warren, 1894, Novit. zool., 1: 407. Type species: Ephyra splendens Butler, 1878, by original designation.

#### Generic characters.

Antenna filiform, ciliate in both sexes. Frons narrow, not protruding. Labial palpi narrow and short. Apex of forewing rounded; outer margin of both wings smooth, or slightly rounded. Forewing length: 7–13 mm. Wings usually pale yellow to yellow, with black or greyish brown markings. Forewing with Sc free, R_1_ coincident with R_2_, R_3+4_ stalked with R_5_, R_3–5_ arising before anterior angle of cell, CuA_1_ arising from or before posterior angle of cell; hindwing with Sc+R_1_ close to cell less than one half of length of cell; Rs arising before anterior angle of cell; CuA_1_ arising from or before posterior angle of cell. An elongate, unscaled, translucent fovea present in male. Male genitalia with uncus almost triangular; median process of gnathos small; valva with a narrow and strongly curved dorsal arm at valva base, with a tiny spine apically; juxta short; saccus broad and rounded; aedeagus short and narrow; vesica often with a sclerotized lobe and a patch of tiny spines. Female genitalia with papillae anales not elongate, slightly narrower terminally; antrum well sclerotized; ductus bursae short; ductus seminalis often with a sclerotized base, and separate from posterior part of corpus bursae; corpus bursae sclerotized and with longitudinal ribbing posteriorly; signum rounded, with long marginal and smaller central spines.

#### Distribution.

China, Japan, Korean Peninsula, (Papua New Guinea, see below).

#### Remarks.

Only one female specimen (the holotype) of *N. flavimedia* was located in NHM. Close examination of the holotype (Fig. 9) revealed that *N. flavimedia* is quite different from its present congeners in both wing patterns and female genitalia. Further morphological study of *Ninodes* and its related genera is needed to determine the correct taxonomic position of *N. flavimedia*. Characters of *N. flavimedia* are not included in the generic description above.

One male and one female probably belonging to *N. watanabei* are kept in the collection of ZFMK (collected at “Tapaishan”, Shaanxi). Before recording this species as new to the Chinese fauna, the specimens must be checked closely and the identity proved by dissection (Dieter Stüning, pers. comm.).

*Ninodes miegi* Sterneck, 1931 has been treated as a junior synonym of *N. splendens* since Inoue (1956). However, we found that the wing pattern of the holotype of *N. miegi* (Fig. 6) is quite similar to *N. albarius* (Figs 4, 5). The above two species were both originally described from Korea. We suspect that *N. miegi* Sterneck, 1931 may be a senior synonym of *N. albarius* and probably has to be restored from synonymy of *N. splendens*. The female holotype of *N. miegi* has to be checked carefully. We will discuss this taxonomic problem in the future revision of *Ninodes*.

*Ninodes scintillans* Thierry-Mieg, 1915 was described as a distinct species, but listed as a junior synonym of *N. splendens* by Scoble (1999). After examining the type of *N. splendens* at BMNH, a photo of a syntype of *N. scintillans* (coll. MNHN) and specimens of both taxa in IZCAS, we found that the male and female genitalia of them are in fact identical (Figs 16–18, 22, 23), although the wing patterns of *N. splendens* (Figs 7, 8) and *N. scintillans* (Figs 10–12) are quite different, as described by Prout (1915) and Wehrli (1939). The identical genitalia are probably the reason why Scoble (1999) listed *N. scintillans* as a junior synonym of *N. splendens*. However, the genitalia of the syntypes of *N. scintillans* have not been studied so far. Examination of the collecting localities and data of the Chinese specimens in IZCAS and ZFMK revealed that *N. splendens* and *N. scintillans* are probably just seasonal forms of the same species, with typical *N. splendens* being the first generation and *N. scintillans* the second. However, further morphological, biological, and molecular studies are needed to test this hypothesis. We will also discuss this problem in the future revision of *Ninodes*.

### Ninodes quadratus sp. n.

Taxon classificationAnimaliaLepidopteraGeometridae

http://zoobank.org/09828123-6308-415C-9E52-801B86921A1B

**Figures 1–12.**
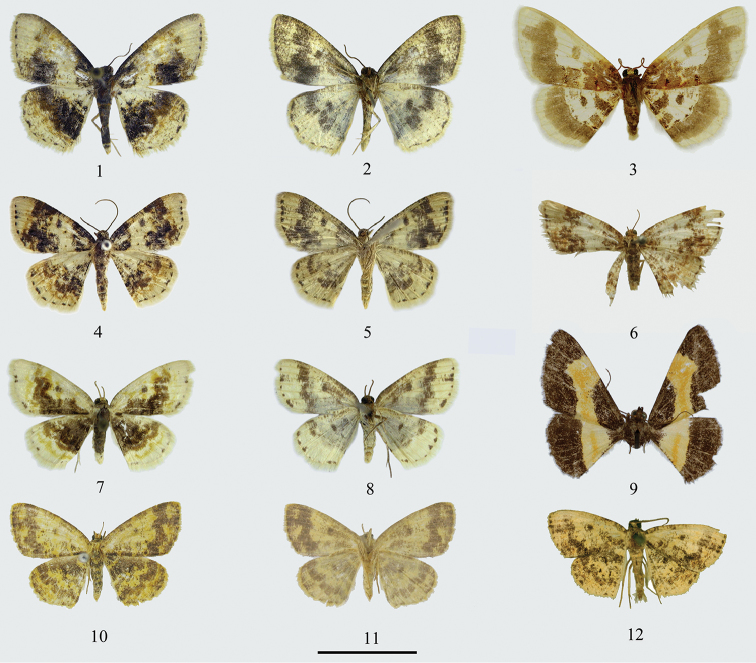
Adults of *Ninodes*. **1–2** *N. quadratus* sp. n. **1** male (holotype) **2** ditto, underside **3** *N. watanabei*, male (holotype, coll. NHM) **4–5** *N. albarius* **4** male **5** ditto, underside **6** *N. miegi*, female (holotype, coll. SMTD) **7–8** *N. splendens* **7** male collected from Shanghai, May **8** ditto, underside **9** *N. flavimedia*, female (holotype, coll. NHM) **10–12** *N. splendens* (“*scintillans*”) **10** male, collected from Gansu in July **11** ditto, underside **12** male, collected from shanghai (syntype of *N. scintillans*, coll. MNHN). Scale bar 1 cm.

**Figures 13–18.**
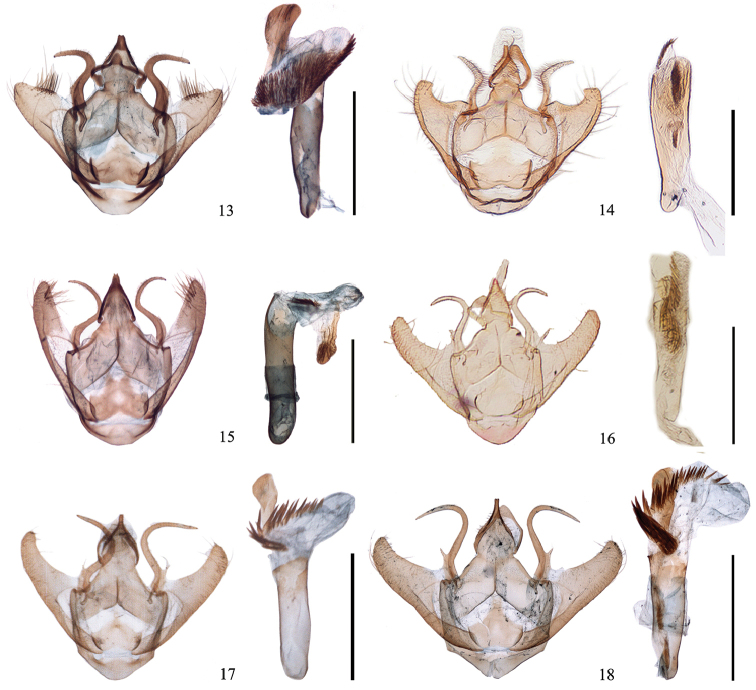
Male genitalia of *Ninodes*. **13** *N. quadratus* sp. n. (paratype, IZCAS slide no. Geom-04014) **14** *N. watanabei* (NHM Inoue slide no. 05182). **15** *N. albarius* (IZCAS slide no. Geom-04015) **16–18** *N. splendens* **16** collected from Japan, (syntype, NHM Geometridae slide no. 07899) **17** collected from Shanghai in May (IZCAS slide no. Geom-04311) **18** collected from Gansu in July (“*scintillans*”) (IZCAS slide no. Geom-02339). Scale bars 1 mm.

**Figures 19–24.**
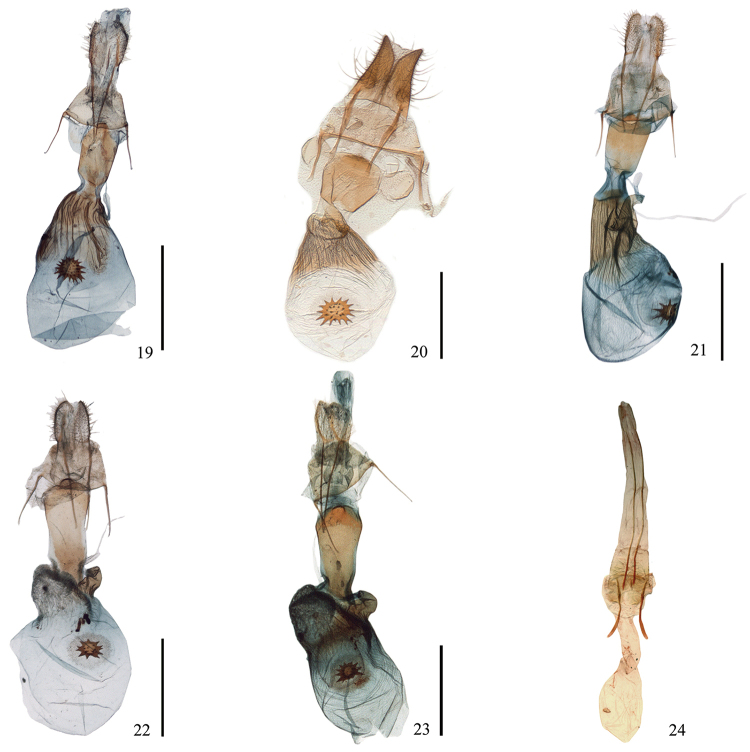
Female genitalia of *Ninodes*. **19** *N. quadratus* sp. n. (paratype, IZCAS slide no. Geom-04005) **20** *N. watanabei* (NHM Inoue slide no. 05183) **21** *N. albarius* (IZCAS slide no. Geom-04046); **22–23** *N. splendens* **22** collected from Shanghai in June (IZCAS slide no. Geom-04052) **23** collected from Hubei in July (“*scintillans*”) (IZCAS slide no. Geom-02966) **24** *N. flavimedia* (holotype, NHM Geometridae slide no. 7901, without scale). Scale bars 1 mm.

Figs 1 , 2 , 13 , 19

#### Description.

**Head.** Antenna filiform, ciliate in both sexes (ciliae of males longer), with mixed pale yellow mixed and black scales dorsally. Frons narrow, black, not protruding. Labial palpus narrow, scaled black. Vertex with large and black scales.

**Thorax**. Patagia, tegulae and dorsal side of thorax black. Hind tibia with two pairs of spurs in both sexes, without hair-pencil in male. Forewing length: 9–10 mm. Apex of forewing rounded; outer margin of forewing only slightly oblique, evenly curved outwards. Ground colour of wings pale yellow. Forewing black from base to antemedial line; discal spot pale grey, indistinct; medial and postmedial line yellow, narrow and wavy; a black quadrate patch present near tornus; terminal line present as a series of short black streaks between veins; fringe pale yellow. Hindwing pale yellow at base, discal spot hardly visible; a broad black band present between medial and postmedial line; submarginal line yellow and wavy, more distinct than that of forewing; terminal line and fringe similar to those of forewing. Underside with discal spot of forewing more distinct than that on upperside; black postmedial bands present on both wings, that of forewing elongate, not quadrate as on upperside, that of hindwing narrower than on upperside, other pattern elements less distinct than those on upperside. Wing venation and fovea of male as mentioned in generic characters.

**Abdomen**. Abdomen dorsally suffused with black scales. Setal comb absent on male sternite III.

**Male genitalia**. Uncus almost triangular, strongly sclerotized and shallowly bifurcate at apex. Gnathos with a small median process, rounded terminally. Valva short, elongate-triangular, narrow and rounded terminally, with a sclerotized ridge, evenly curved outwards, costal margin curved outwards, with a row of long and spine-like setae medially. A well-developed dorsal arm present at valva base, strongly curved to S-shape, with a tiny spine apically and a long, curved, rod-like basal process. Juxta short and broad, tapering terminally. Saccus broad and rounded. Aedeagus short and narrow; vesica with a large spatulate lobe and a large dense patch of of tiny spines, laterally extended into a row of short, stout spines.

**Female genitalia**. Papillae anales not elongate, slightly narrower terminally. Apophyses anteriores about 3/5 length of apophyses posteriores. Antrum well sclerotized, about one half length of corpus bursae. Ductus bursae very short. Ductus seminalis with a small sclerotized base, separated from posterior part of corpus bursae. Corpus bursae sclerotized and with longitudinal ribbing at posterior half; signum on ventral side, rounded, margins and internal surface covered with spines.

#### Diagnosis.

Concerning wing patterns, the new species can be distinguished from the other congeners by the broad black quadrate patch at medial to postmedial area of the forewing, becoming indistinct above CuA_2_; the discal spot of the forewing is more distinct on the underside than that on the upperside. In the male genitalia, the new species has a distinctive shape of valva and arrangement of cornuti: the costal margin of the valva is curved outwards medially, while it is almost straight in *N. albarius* (Fig. 15), slightly curved inwards in *N. splendens* (Figs 16–18), and forming a semicircular cleft in *N. watanabei* (Fig. 14); the spatulate lobe of the vesica is similar to that of *N. splendens*, but larger and the large dense patch of small spines present in *N. quadratus* is rather arranged in a longitudinal line of much larger spines in *N. splendens* and a very short row of small spines in *N. albarius*. The lateral row of short stout spines is absent in *N. splendens* and *N. albarius*. The female genitalia of the new species are similar to *N. albarius*, but the base of the ductus seminalis is almost conic and separated from the corpus bursae in *N. albarius* (Fig. 21), while this character is absent in the new species. The antrum is similar in length to *N. albarius*, but much shorter than that of *N. splendens* (Figs 22, 23), and is much narrower compared to *N. watanabei* (Fig. 20). The signum of *N. quadratus* is larger and has more but smaller marginal spines than those of *N. splendens* and *N. albarius*.

#### Material examined.

Holotype, ♂, **CHINA: Henan** (IZCAS): Baotianman, 623 m, 12.VIII.2008, coll. Xue Dayong. Paratypes: 1♂, same data as holotype. **Henan** (IZCAS): 1♂, Haoxian, Baiyunshan, 115 m, 7–9.VIII.2013, coll. Jiang Nan. **Shaanxi **(IZCAS): 5♂2♀, Shangnan, Jinsixia, 777 m, 23–25.VII.2013, coll. Cui Le & Jiang Nan; 3♂, Ningshan, Guanghuojie, Baohuzhan, 1081–1189 m, 26–28.VII.2014, coll. Liu Shuxian & Ban Xiaoshuang. **Gansu** (IZCAS): 1♂, Wenxian, Qiujiaba, 2200–2300 m, 16–19.VII.2003, coll. Wang Hongjian; 4♂5♀, Wenxian, VI–IX.2002, coll. Wang Hongjian; 1♂, Bikou, Baohuzhan, 645 m, 6–7.VIII.2016, coll. Cheng Rui & Jiang Shan; 5♂1♀, Bikou, Bifenggou, 720 m, 8–10.VIII.2016, coll. Cheng Rui & Jiang Shan. **Zhejiang** (ZFMK): 1♂, West-Tien-Mu-Shan, 1600 m, 14.IV.1932, coll. H. Höne.

#### Distribution.

China (Henan, Shaanxi, Gansu, Zhejiang).

#### Etymology.

The specific name refers to the quadrate (Latin: *quadratus*) black patch near the tornus of the forewing.

### Ninodes albarius

Taxon classificationAnimaliaLepidopteraGeometridae

Beljaev & Park, 1998

Figs 4 , 5 , 15 , 21

Ninodes albarius Beljaev & Park, 1998, Tinea, 15 (3): 243, figs 1, 4, 13. Holotype ♂, Gwangleung, Korea (NIAST).

#### Diagnosis.

*N. albarius* is similar to *N. splendens* externally, but can be distinguished by the following features: a broad, black band, reaching from costa to anal margin, is present at the base of the forewing, while in *N. splendens*, it is absent or only present near the anal margin; in the male genitalia, the valvae are longer and narrower, more strongly pointed apically and with a group of strong spines near apex, the terminal part of the juxta is broader and incised, the cornuti are smaller and arranged in a short band. In the female genitalia, the antrum is shorter, the posterior part of the corpus bursae is narrower and more strongly sclerotized, and the signum is larger in *N. albarius*. By the wing patterns, *N. albarius* also resembles *N. quadratus* and *N. watanabei* in the basal black or greyish brown band, but it is different from *N. quadratus* by the absence of the quadrate patch near the tornus and the distinct distal spot on the upperside of the forewing, and differs from *N. watanabei* by the smaller size and the absence of the protrusion on the postmedial band between veins M_1_ and M_3_ of the forewing.

#### Material examined.

**CHINA: Henan** (IZCAS): 1♂, Tongbai, Shuiliandong, 300 m, coll. Shen Xiaocheng and Ren Yingdang; 2♂, Xinyang, Jigongshan, 250 m, 20–21.VII.2002, coll. Han Hongxiang. **Shaanxi** (IZCAS): 1♂, Zhouzhi, Houzhenzi, 1350 m, 24.VI.1999, coll. Zhu Chaodong; 2♂1♀, Shangnan, Jinsixia, 777 m, 23–25.VII.2013, coll. Cui Le & Jiang Nan; 1♂, Xunyang, Jinxinyuan, Shanzhuang, 386 m, 1–3.VIII.2014, coll. Ban Xiaoshuang. **Gansu** (IZCAS): 3♂, Bikou, Baohuzhan, 645 m, 6–11.VIII.2016, coll. Cheng Rui & Jiang Shan; 2♂1♀, Bikou, Bifenggou, 720 m, 8–10.VIII.2016, coll. Cheng Rui & Jiang Shan. **Hubei** (IZCAS): 11♂1♀, Yingshan, Wujiashan, 860 m, 28–30.VI.2014, coll. Cui Le, Jiang Nan & Xue Dayong; 3♂1♀, Yingshan, Wujiashan, Jingqu, 500 m, 30.VI.2015, coll. Xue Dayong; 10♂, Luotian, Qingtaiguan, 560 m, 1–4.VII.2014, coll. Cui Le, Jiang Nan & Xue Dayong; 1♂, Yunxi, Guanyinzhen, 289–305 m, coll. Ban Xiaoshuang.

#### Distribution.

Korean peninsula and China (Henan, Shaanxi, Gansu, Hubei); this is a new record for China.

## Supplementary Material

XML Treatment for Ninodes

XML Treatment for Ninodes quadratus

XML Treatment for Ninodes albarius
